# Nature's Poison Pill: A Case of Digoxin-Like Toxicity From the Seed of the Ornamental Plant Thevetia peruviana

**DOI:** 10.7759/cureus.112917

**Published:** 2026-07-18

**Authors:** Krupa A Sunil, Karl-Anthony Robinson, Jacqueline Milton-Brown

**Affiliations:** 1 Pharmacy, Harris Health System, Houston, USA

**Keywords:** cardiac glycosides, digoxin, digoxin immune fab, poisoning, thevetia peruviana, yellow oleander

## Abstract

*Thevetia peruviana *(yellow oleander) is an ornamental shrub found in tropical and subtropical regions, including tropical America. All parts of the plant are toxic due to cardiac glycosides, which can cause severe cardiotoxicity and arrhythmias. Poisoning is associated with significant mortality, often due to deliberate self-harm. This report describes a case of accidental ingestion of *Thevetia peruviana* seeds by a patient who believed that they could treat benign prostatic hyperplasia (BPH). The patient presented to the emergency department within 30 minutes of ingesting yellow oleander seeds with nausea, vomiting, and abdominal cramps. Although initial vital signs were stable, he developed sinus bradycardia and symptoms of cardiac glycoside toxicity, including dizziness, tachypnea, and visual disturbances. Lab results later showed a serum digoxin level of 4.04 ng/mL, and the poison control center recommended digoxin immune fab (DigiFab). He received a total of 15 vials intravenously, resulting in marked clinical improvement. He was monitored on continuous telemetry in the cardiac unit and discharged in stable condition after four days. Although DigiFab is not approved by the U.S. Food and Drug Administration for oleander poisoning, it remains the most effective reported antidote, and timely administration is crucial for successful management. Given the limited reports of oleander toxicity in the United States, this case highlights the risks of unregulated herbal remedies and the clinical presentation and management of oleander toxicity, underscoring the need for public education about oleander toxicity and the dangers of unregulated herbal remedies.

## Introduction

*Thevetia peruviana* (yellow oleander) is a shrub in the Dogbane family “Apocynaceae,” commonly found in tropical and subtropical areas including tropical America. Poisoning due to deliberate self-harm (DSH) results in significant mortality rates in South Asian countries, especially India and Sri Lanka. In Sri Lanka, most cases were reported around 2005-2010, when there was a wave of DSH attempts [1]. Ingestion of any part of this plant is toxic because it contains cardiac glycosides including thevetin A, thevetin B, neriifolin, and oleandrin that can cause cardiotoxicity and fatal arrhythmias [2]. *Thevetia peruviana* contains cardiac glycosides that inhibit the sodium-potassium ATPase pump in myocardial cells, increasing intracellular sodium and promoting calcium influx via the sodium-calcium exchanger. This enhances myocardial contractility while increasing vagal tone, slowing cardiac conduction, decreasing chronotropy, and prolonging the refractory period [1-3]. The first-line treatment for severe cardiac glycoside toxicity is digoxin immune fab (DigiFab). It rapidly binds to free digoxin with high affinity, thereby dissociating digoxin from sodium/potassium ATPase pumps [4,5]. While this plant grows widely across America, fatal poisoning remains uncommon, underscoring the importance of awareness of its toxicity. This case report highlights the toxic effects of *Thevetia peruviana*, the symptoms associated with its poisoning, and the importance of timely administration of DigiFab, which can be lifesaving. It also raises public awareness about the commercial availability of these seeds and the risks associated with consuming commercially available products that lack proper safety and efficacy validation.

## Case presentation

**Figure 1:**
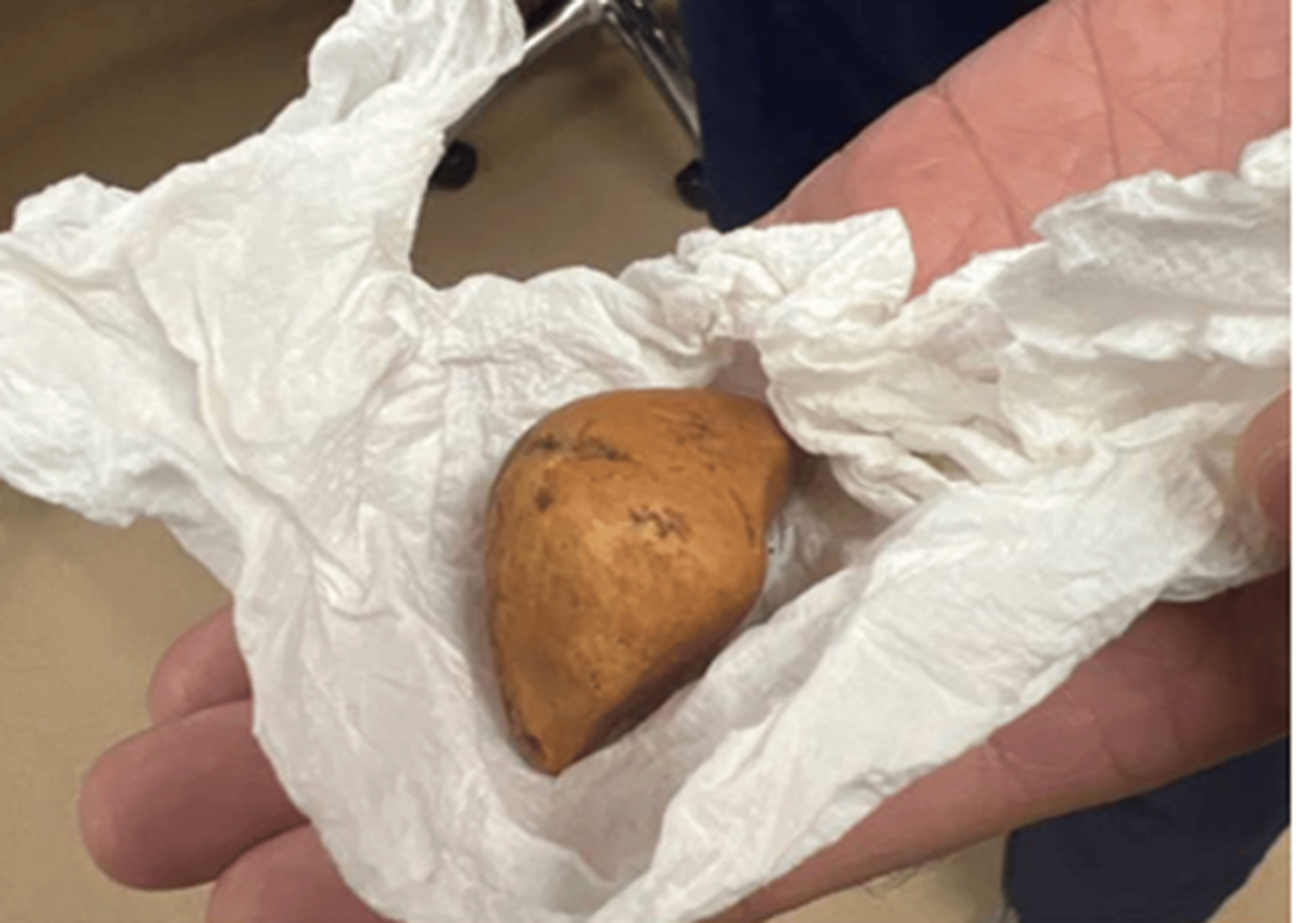
Photograph of the ingested “lucky nut” provided by the family

This case involves a 70-year-old male with a medical history significant for benign prostatic hyperplasia (BPH), hypertension, hyperlipidemia, lung nodules, prediabetes, and subclinical hypothyroidism. His home medications include gabapentin, doxazosin, levothyroxine, atorvastatin, finasteride, and aspirin. As part of his self-directed homeopathic practices, he presented after ingesting *Thevetia peruviana*, which he mistook for a Brazilian nut (commonly referred to as a “lucky nut”) (refer to Figure 1 for the seed purchased by the patient). Within 30 minutes of ingestion, he was brought to the hospital, where he subsequently developed nausea, vomiting, and abdominal cramps and denied any dyspnea, syncope, or chest pain. Tables 1, 2 show his initial vitals and labs. He subsequently developed multiple episodes of sinus bradycardia (40 to 50 beats per minute [bpm]). There was no murmur or gallop on auscultation, and the patient was alert and oriented with a Glasgow Coma Scale (GCS) score of 15. The poison control center was consulted, which recommended administering five vials of DigiFab intravenously every 10 minutes, up to a maximum of 15 vials.

**Table 1:** Initial vitals at the Emergency Department

Parameters	Values	Reference Range
Blood pressure	112/68 mm Hg	120/80 mmHg
Heart rate	65 bpm	60-100 bpm
Temperature	98.4°F	97-99°F
Respiratory rate	23 breaths per minute	12-20 breaths per minute
Oxygen saturation	99% on ambient air	95%-100%

**Table 2:** Lab values BUN, blood urea nitrogen

Parameters	Values	Reference Range
Potassium	4.5 mmol/L	3.5-5.2 mmol/L
Magnesium	1.8 mg/dL	1.7-2.2 mg/dL
Calcium	9.8 mg/dL	8.6-10.3 mg/dl
Sodium	137 mmol/L	136-145 mmol/L
Serum digoxin	4.04 ng/mL	0.90-2 ng/mL
Serum creatinine	1.1 mg/dL	0.7-1.3 mg/dL
BUN	12 mg/dL	7-25 mg/dL

The patient was given four vials of available intravenous (IV) DigiFab but remained in sinus bradycardia with a heart rate (HR) of 40 bpm and a blood pressure (BP) ranging from 125-135/63-75 mmHg secondary to digoxin-like toxicity. Additionally, 4 g of magnesium sulfate was administered for its antiarrhythmic effect. He was transferred to the Cardiac Care Unit after developing dizziness, tachypnea, and reported visual disturbances (dark-brown and orange hues) along with jerking movements, facial twitching, and periods of unresponsiveness. Additionally, the patient experienced worsening bradycardia at 30 bpm and a BP of 112/62 mmHg. A dose of 1 mg of atropine increased the HR to 120 bpm. The patient became responsive but still reported blurry vision. He denied chest pain, numbness, tingling, or shortness of breath. However, he developed hypothermia, which was managed with a Bair Hugger device (3M, St. Paul, MN, USA).

**Figure 2:**
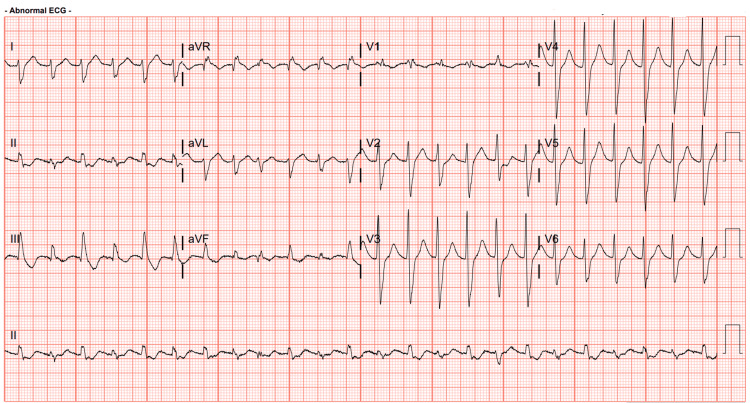
Atrial fibrillation with rapid ventricular response with aberrant conduction or ventricular complexes (ECG obtained during DigiFab therapy).

DigiFab therapy was re-initiated. He experienced multiple arrhythmias, including sinus bradycardia, variable type II atrioventricular (AV) blocks, and episodes of nausea. He received a total of 8 vials of IV DigiFab on the first day of admission for persistent atrial fibrillation with AV block and bradycardia (Figure 2).

Overall, he received 15 vials (600 mg) of DigiFab within the first two days. The total serum digoxin level decreased to 0.99 ng/mL. After early bradycardic episodes requiring intervention, his HR stabilized to 60 bpm and BP ranged from 100-120/57-71 mmHg without further intervention. His symptoms resolved, and he was discharged in stable condition after four days of hospitalization. At the one-week follow-up, he remained hemodynamically stable and had returned to his baseline functional status.

## Discussion

*Thevetia peruviana* grows in different parts of the world and contains cardiac glycosides that inhibit the sodium/potassium ATPase pump in myocardial cells. This raises intracellular sodium, leading to increased calcium influx via the sodium-calcium exchange pump, and stronger myocyte contraction. It also increases vagal tone, resulting in decreased chronotropy, and slows conduction and prolongs the refractory period in cardiac tissue. At toxic concentrations, these effects disrupt electrical stability, causing arrhythmias due to increased cell excitability and decreased resting potential [1-3].

Acute ingestion typically causes vomiting, dizziness, abdominal pain, dilated pupils, and drowsiness. Patients may experience hyperkalemia and arrhythmias, most commonly bradyarrhythmias and AV blocks. Similar results were found in a retrospective observational study, where patients presented to the hospital at a mean of 12.29 ± 8.48 hours after ingestion of yellow oleander, with severity ranging from vomiting (80%) to death (6.7%) [6]. All parts of the plant are toxic, with severity depending on the amount ingested and pharmacokinetics.

The usual toxic dose is 15 to 20 g of roots or 8 to 10 seeds, though this varies depending on the concentration, crushing of the plant, individual response, and potential comorbidities. Signs and symptoms of toxicity may be delayed due to the median half-life of approximately 42.9 hours [7].

First-line treatment for severe cardiac glycoside toxicity is DigiFab. It binds free digoxin with high affinity, removing it from Na⁺/K⁺- ATPase receptors. Approximately 40 mg of DigiFab neutralizes 0.5 mg of digoxin [4,5]. Administration may cause a rapid drop in serum potassium, necessitating frequent monitoring, especially early after administration [7,8]. For acute overdose, activated charcoal may be used if gastrointestinal absorption is ongoing [8,9]. A single dose of activated charcoal is most effective within 1 hour of toxin ingestion, at a dose of 50-100 g, while multi-dose activated charcoal may be used for agents with prolonged absorption or enterohepatic recirculation, at 50 g every 4-6 hours [2,5,8]. Administration of activated charcoal beyond 1 hour after ingestion may still be beneficial in selected clinical scenarios, such as ingestion of extended-release formulations, substances that delay gastric emptying, or massive overdoses in which ongoing gastrointestinal absorption is expected [6,10]. Charcoal reduces toxicity by limiting absorption and interrupting enterohepatic recirculation, increasing drug clearance from 12 L/h to 18 L/h, and reducing the half-life from 37 to 22 hours [9]. Aggressive fluid resuscitation and correction of electrolyte imbalances form the mainstay of supportive treatment in yellow oleander poisoning [7,9].

Although herbal products are widely perceived as safe because of their natural origin, they may pose significant health risks despite being marketed as beneficial. The patient in this case consumed the *Thevetia peruviana* seed believing it would alleviate his BPH but instead suffered life-threatening toxicity. While this plant grows widely across America, fatal poisoning remains uncommon, underscoring the importance of awareness of its toxicity.

This case report is limited by its single-patient design, restricting generalizability. Additionally, limited access to DigiFab in resource-limited settings may hinder treatment, and without a comparator, the efficacy and optimal dosing in yellow oleander poisoning cannot be confirmed.

## Conclusions

This case report highlights the importance of early recognition of yellow oleander poisoning and the prompt administration of DigiFab, which proved lifesaving in a patient who ingested a *Thevetia peruviana *seed. It also underscores the need for greater public awareness of the potential dangers associated with unregulated herbal remedies. Furthermore, this case emphasizes that products marketed as natural are not inherently safe and reinforces the importance of seeking guidance from healthcare professionals before using such remedies.
